# Association between mitochondrial DNA variations and schizophrenia in the northern Chinese Han population

**DOI:** 10.1371/journal.pone.0182769

**Published:** 2017-08-28

**Authors:** Feng-ling Xu, Mei Ding, Jun Yao, Zhang-sen Shi, Xue Wu, Jing-jing Zhang, Hao Pang, Jia-xin Xing, Jin-feng Xuan, Bao-jie Wang

**Affiliations:** School of Forensic Medicine, China Medical University, Shenyang, China; San Francisco Coordinating Center, UNITED STATES

## Abstract

To determine whether mitochondrial DNA (mtDNA) variations are associated with schizophrenia, 313 patients with schizophrenia and 326 unaffected participants of the northern Chinese Han population were included in a prospective study. Single-nucleotide polymorphisms (SNPs) including C5178A, A10398G, G13708A, and C13928G were analyzed by polymerase chain reaction–restriction fragment length polymorphism (PCR–RFLP). Hypervariable regions I and II (HVSI and HVSII) were analyzed by sequencing. The results showed that the 4 SNPs and 11 haplotypes, composed of the 4 SNPs, did not differ significantly between patient and control groups. No significant association between haplogroups and the risk of schizophrenia was ascertained after Bonferroni correction. Drawing a conclusion, there was no evidence of an association between mtDNA (the 4 SNPs and the control region) and schizophrenia in the northern Chinese Han population.

## Introduction

Schizophrenia is a chronic, severe metal dysfunction. Clinical manifestations of schizophrenia vary tremendously, and the pathogenesis of this disease is unclear. Results of studies in which twins or adopted children were associated with occurrence of schizophrenia indicated that genetics and environmental factors together can produce this disease [1]. It was reported that the rate of mental illness among offspring was higher for those with a maternal history of mental illness than for those with a paternal history [2]. Patients with mitochondrial disease also were more likely to exhibit symptoms of mental illness [3]. Therefore, the risk of schizophrenia might be related to mitochondrial dysfunction.

The coding region of mtDNA encodes 13 proteins, 22 tRNAs, and 2 rRNAs. The control region regulates replication and expression of the mitochondrial genes and harbors a replication initiation site and 2 major transcription initiation sites [4]. Mitochondria dysfunction can severely affect neuronal activity, including synaptic connection, axon formation, and neuronal plasticity [5]. Other investigators determined that variations in mtDNA altered the construction and expression levels of relevant proteins [6]. These variations can yield defects in respiration, enhance glycolysis, and induce overproduction of reactive oxygen species (ROS) [7]. ROS overproduction eventually leaded to neuronal damage [8]. Activity of the oxidative respiratory chain complex I might be an indicator of schizophrenia [9]. To further assess this possibility, the 4 SNPs involved in encoding complex I—C5178A, A10398G, G13708A, and C13928G—were examined in this study.

The variation 5178 C → A in the MT-ND2 gene results in substitution of leucine-237 with methionine in mitochondrial membrane nicotinamide adenine dinucleotide (MT-NADH) dehydrogenase subunit 2 (ND2-Leu237Met) [10]. The alteration of the base at site 5178 might affect the structure and function of the MT-NADH dehydrogenase subunit 2, which in turn can affect oxidative phosphorylation, oxidative stress, and glucose metabolism [11]. Authors have found previously that 5178C → A base substitution could protect mitochondria from oxidative damage [12]. Associations were made between C5178A and longevity [13], Parkinson’s syndrome [14], and bipolar disorder [15].

The mutation 10398A → G in the MT-ND3 gene leads to substitution of threonine-114 with alanine in MT-NADH dehydrogenase subunit 3 (MT-ND3-Thr114Ala). Investigators associated G10398A with a variety of diseases, including bipolar disorder [15], type 2 diabetes [16], and breast cancer [17]. However, results of 1 study did not find an association between G10398A of mtDNA and breast cancer in the Chinese Han population [18]. Moreover, no relationship was found between the G10398A polymorphism and Alzheimer’s disease, Parkinsonism, or migraine [19].

Both G13708A and C13928G polymorphisms are in the MT-ND5 gene. The 13708G → A replacement results in substitution of alanine-458 with threonine in MT-NADH dehydrogenase subunit 5 on the mitochondrial membrane (MT-ND5-Ala458Thr). It was found that the 13708G → A polymorphism was probably not related to Alzheimer’s disease [20], Parkinson’s disease, or migraine [19]. Substitution of 13928C → G results in replacement of threonine-531 with serine in the MT-NADH dehydrogenase subunit 5 on the mitochondrial membrane (MT-ND5-Thr531Ser).

Mutations in these genes can alter the amino acid sequence and affect protein structures and functions [10]. Presently, there are few studies of the relationship between schizophrenia and SNPs (C5178A, A10398G, G13708A, and C13928G) in the northern Han Chinese population or of the relationship between hypervariable regions of mtDNA and schizophrenia.

## Materials and methods

### Samples

This study included venous blood specimens from 326 unrelated healthy individuals (control group) and 313 patients with schizophrenia (patient group) of the northern Han Chinese population (Table 1). Samples were provided by China Medical University. In the control group, individuals (mean age ± standard deviation [SD], 43.7 ± 7.4 years; range, 25–65 years; 162 men, 164 women) were confirmed to be unaffected by mental illness through at least 3 generations. Unaffected control participants also were screened with the Scaled Global Health Questionnaire (GHQ) [21], and individuals with potential psychiatric disease were excluded. Only unrelated participants with no personal history of psychiatric disease and a GHQ score <7 were considered for inclusion in the study [22]. The control group and patient group were matched for ethnicity, age, gender, and geographical region. Patients with schizophrenia (mean age ± SD, 41.1 ± 7.1 years; range, 21–65 years; 157 men, 156 women) had been hospitalized for <1 month and met DSM-IV criteria for the diagnosis of schizophrenia. Patients were evaluated by at least 2 psychiatrists who reached consensus on diagnosis. Participants were recruited from 2005 to 2010. All patients and participants provided written informed consent prior to inclusion in this study. Specimens were obtained and analyzed with approval from the Ethics Committee of China Medical University.

**Table 1:** Characteristics of the study participants.

Variables	Control Group (N = 326)	Patient Group (N = 313)
Age (mean ± SD)	43.7±7.4	41.1±7.1
Male (%)	49.7	50.2
Female (%)	50.3	49.8
Male age (mean ± SD)	47.1±7.9	42.5±6.9
Female age (mean ± SD)	40.5±6.9	39.7±7.2

### Methods

#### DNA extraction, amplification, and sequencing

DNA was extracted from sample blood using the Chelex-100 method. The mtDNA fragment (15869–740) was amplified using primers for polymerase chain reaction (PCR): L15869F and H719R [23] (Table 2). The 20 μl PCR reactions contained 2.0 μl 5×buffer, 1.6 μl 2.5 mM dNTP mix, 0.8 μl each of reverse (R) and forward (F) PCR primers (8 pM each), 0.2 μl of KOD Enzyme (1.0 U/μl), and 20 ng of template DNA. PCR was performed under the following cycle conditions: initial denaturation of 94°C for 5 minutes; followed by 35 cycles of 94°C denaturation for 30 seconds, 55°C annealing for 30 seconds, and 72°C elongation for 40 seconds; followed by a final extension at 72°C for 10 minutes. The production fragment was sequenced with the following primers: L15869F and 80R for HVSI, 16539F and H719R for HVSII. The purified PCR products were sequenced by ABI 377 DNA automatic sequencer. We deposited the laboratory protocols in protocols.io (dx.doi.org/10.17504/protocols.io.ipccdiw).

**Table 2:** Primers used for the analysis of mtDNA polymorphisms in the hypervariable region and the coding region.

Locus	Annealing Temperature (°C)	Primer Sequences (5ʹ → 3ʹ)
Hypervariable		L15869 F 5' AAAATACTCAAATGGGCCTGTC 3'
Regions		
		H719 R 5' CGTGGTGATTTAGAGGGTGAAC 3'
		16539 F 5' ACACGTTCCCCTTAAATAAGAC 3'
		80 R 5' AGCGTCTCGCAATGCTATCG 3'
5178	61°C	5178 F 5' ATCTCTCCCTCACTAAACGTAAGCCTT 3'
		5178 R 5' TTAGTATAAAAGGGGAGATAGGTAGGAGTAGC 3'
10398	64°C	10398 F 5' GCCCTCCTTTTACCCCTACCA 3'
		10398 R 5' GGGAGGATATGAGGTGTGAGCGAT 3'
13708	65°C	13708 F 5' TCATCGCTACCTCCCTGACAAG 3'
		13708 R 5' ATGCTAGGGTAGAATCCGAGTATGTT 3'
13928	61°C	13928 F 5' TATTCGCAGGATTTCTCATTACTAACAACATTTC 3'
		13928 R 5' AAAATATATAAGGATTGTGCGGTGTGTGACG 3' ^a^

^a^ Mismatched base.

#### PCR amplification and restriction fragment length polymorphism analysis of the mtDNA coding region

The four SNPs (C5178A, A10398G, G13708A, and C13928G) in the mtDNA coding region were detected using PCR-RFLP analysis. The primers listed in able 2 were used to amplify target fragments. The mismatch method was applied to generate an HpyCH4Ⅲ artificial restriction endonuclease site in the amplified fragment that included the C13928G SNP. The 20 μl PCR reactions contained 2.0 μl 10×buffer, 2 μl 2.5 mM dNTP mix, 2 μl each of R and F PCR primers (10 pM each), 0.2 μl of rTaq Enzyme (5 U/μl), and 20 ng of template DNA. PCR was performed under the following cycle conditions: initial denaturation of 94°C for 5 minutes; followed by 30 cycles of 94°C denaturation for 30 seconds, annealing at 61–65°C (Table 2) for 30 seconds, and elongation at 72°C for 30 seconds; followed by a final extension at 72°C for 5 minutes. PCR products were digested with restriction enzymes (S1 Table), and fragments were detected on 6% polyacrylamide gel.

#### Data analysis

The purified PCR products were sequenced by ABI 377 DNA automatic sequencer. Sample sequences were validated twice with Sequencher 4.1.4 software (Gene Codes Corp, Ann Arbor, MI, USA) to ensure accuracy of the data set. The sequences then were aligned and compared with rCRS [24]. The mtDNA haplogroups were classified according to the mtDNA Build16 (19 February 2014) using MitoTool [25]. Differences between the control and patient groups were ascertained using SPSS PASW Statistics v. 18.0 (IBM, Chicago, IL). The significance threshold for the haplogroup tests was 0.0020 (0.05/25); 0.0022 (0.05/22) for females and 0.0021 (0.05/24) for males [26]. Power analysis was conducted by PS program [27] statistical software.

#### Access to data

Sequences derived in this study have been deposited into the NCBI database under the following accession numbers: KY212209-KY212525, KY432960-KY432968, and KY432969-KY433281 (https://www.ncbi.nlm.nih.gov/genbank/). Accession numbers of control-group sequences are KY212209-KY212525 and KY432960-KY432968; accession numbers of patient-group are KY432969-KY433281.

## Results

### No association between the 4 SNPs in the coding region and schizophrenia

Based on sequences of the hypervariable regions and PCR-RFLP fragments in the coding region, the 326 samples in the control group were divided into 312 haplotypes; the 313 samples in the patient group were divided into 301 haplotypes (S1 and S2 Tables). Allelic distributions of the 4 SNPs in the patient and control groups were summarized in Table 3. In the northern Chinese Han population, the minor frequencies of C5178A, A10398G, G13708A, and C13928G were 0.255, 0.472, 0.058, and 0.123, respectively. These substitutions have been reported in approximately 2700 mtDNA sequences at rates of 0.111, 0.541, 0.064, and 0.033, respectively [28]. There were no significant between-group differences in allele frequencies of the 4 SNPs (Table 4). Genotype frequencies of the 4 SNPs also were not significantly different between the control and patient groups (Table 5).

**Table 3:** Allelic distributions of each SNP in patient and control groups.

	Total Number (%)		Female Number (%)		Male Number (%)	
SNP Locus	Patients	Controls	Patients	Controls	Patients	Controls
5178A	90 (28.75)	83 (25.46)	48 (30.77)	37 (22.56)	42(26.75)	46 (28.40)
5178C	223 (71.25)	243 (74.54)	108 (69.23)	127 (77.44)	115 (73.25)	116 (71.60)
10398A	135 (43.13)	154 (47.24)	67 (42.95)	82 (50.00)	68 (43.31)	72 (44.44)
10398G	178 (56.87)	172 (52.76)	89 (57.05)	82 (50.00)	89 (56.69)	90 (55.56)
13708A	16 (5.11)	19 (5.83)	6 (3.85)	9 (5.49)	10 (6.37)	10 (6.17)
13708G	297 (94.89)	307 (94.17)	150 (96.15)	155 (94.51)	147 (93.63)	152 (93.83)
13928C	44 (14.06)	40 (12.27)	22 (14.10)	21 (12.80)	22 (14.01)	19 (11.73)
13928G	269 (85.94)	286 (87.73)	134 (85.90)	143 (87.20)	135 (85.99)	143 (88.27)

**Table 4:** Associations of the 4 SNPs with schizophrenia in the northern Chinese Han population (significance level = 0.05).

	Associated Allele	5178A	10398A	13708A	13928C
Total	*P*	0.349	0.297	0.691	0.504
	*OR*	1.182	0.847	0.870	1.170
	95% *CI*	0.833–1.676	0.620–1.157	0.439–1.725	0.739–1.852
	*Power*	0.155	0.181	0.068	0.103
Female	*P*	0.097	0.206	0.487	0.734
	*OR*	1.526	0.753	0.689	1.118
	95% *CI*	0.926–2.514	0.485–1.170	0.239–1.983	0.588–2.126
	*Power*	0.383	0.243	0.105	0.063
Male	*P*	0.743	0.839	0.942	0.542
	*OR*	0.921	0.955	1.034	1.227
	95% *CI*	0.563–1.505	0.614–1.486	0.418–2.557	0.636–2.367
	*Power*	0.062	0.055	0.051	0.094

**Table 5:** Relationship between haplotypes comprising the 4 SNPs and schizophrenia (significance level = 0.05).

C5178A	A10398G	G13708A	C13928G	Patients	Controls	*P*	*Power*
A	A	A	G	0	3	0.262	0.159
A	A	G	G	24	21	0.545	0.093
A	G	A	G	0	1	1.000	0.086
A	G	G	G	66	58	0.292	0.184
C	A	A	C	12	11	0.755	0.062
C	A	A	G	3	2	0.964	0.079
C	A	G	C	30	27	0.564	0.089
C	A	G	G	66	90	0.055	0.482
C	G	A	G	1	1	1.000	0.050
C	G	G	C	2	2	1.000	0.050
C	G	G	G	109	110	0.773	0.059

### No association of haplogroups with schizophrenia

As shown in Table 6, the frequency of haplogroup M8a significantly differed between groups (*P* = 0.019, OR = 0.315 [0.114–0.869]). Among females, a statistically significant difference was detected in the frequency of haplogroup A between the 2 groups (*P* = 0.043, OR = 0.465 [0.219–0.989]) (Table 7). As depicted in Table 8, the frequencies of haplogroups M7 and N9 were significantly different between male patients with schizophrenia and unaffected male participants (*P* = 0.028, OR = 3.074 [1.080–8.749]; *P* = 0.040, OR = 0.348 [0.122–0.990], respectively). However, the significance could not survive the Bonferroni correction. Therefore, we did not establish an association between haplogroups and schizophrenia in the northern Chinese Han population.

**Table 6:** Relationship between haplogroups and schizophrenia (significance level = 0.05/25).

Haplogroups	Patients (313)	Controls (326)	*P*	*OR*	*95% CI*	*Power*
**A**	26	38	0.159	0.687	0.406–1.161	0.290
**B**	39	47	0.469	0.845	0.535–1.333	0.111
B4	28	34	0.526	0.844	0.499–1.428	0.096
B5	11	13	0.753	0.877	0.387–1.988	0.127
**M8**	28	40	0.173	0.702	0.422–1.170	0.276
M8a	5	16	**0.019**	0.315	0.114–0.869	0.651
C	11	16	0.381	0.706	0.322–1.545	0.140
Z	11	8	0.430	1.448	0.575–3.648	0.126
CZ	1	0	0.490	0.997	0.991–1.003	─
**D**	67	66	0.718	1.073	0.732–1.572	0.065
**R9**	46	36	0.167	1.388	0.870–2.213	0.281
F	46	34	0.103	1.480	0.922–2.375	0.370
**G**	15	12	0.485	1.317	0.607–2.860	0.108
L3*	3	8	0.146	0.385	0.101–1.463	0.304
L3b	3	8	0.146	0.385	0.101–1.463	0.304
M*	16	13	0.495	1.297	0.613–2.743	0.105
**M7**	22	14	0.134	1.685	0.846–3.355	0.323
**M9**	27	19	0.171	1.525	0.830–2.803	0.276
**N9**	15	25	0.133	0.606	0.313–1.172	0.322
N9a	10	19	0.110	0.533	0.244–1.166	0.357
Y	5	6	0.813	0.866	0.262–2.866	0.056
**JT**	3	1	0.587	3.145	0.325–30.398	0.180
R*	1	2	1.000	0.519	0.047–5.755	0.082
**RO**	3	3	1.000	1.042	0.209–5.202	0.050
**others**	2	2	1.000	1.042	0.146–7.442	0.050

* Name of the haplogroup.

**Table 7:** Relationship between haplogroups and schizophrenia among females (significance level = 0.05/22).

Haplogroups		Female Patients (156)	Female Controls (164)	*P*	*OR*	95% *CI*	*Power*
**A**	11		23	**0.043**	0.465	0.219–0.989	0.818
**B**		20	23	0.752	0.902	0.474–1.716	0.073
**M8**		12	23	0.070	0.511	0.245–1.066	0.728
M8a		3	9	0.093	0.338	0.090–1.271	0.663
C		5	11	0.151	0.461	0.156–1.357	0.527
Z		4	3	0.947	1.412	0.311–6.414	0.097
**D**		36	31	0.359	1.287	0.750–2.209	0.254
**R9**		22	20	0.613	1.182	0.617–2.264	0.110
F		22	18	0.398	1.332	0.684–2.591	0.223
**G**		8	3	0.105	2.901	0.755–11.140	0.626
L3*		3	5	0.774	0.624	0.146–2.654	0.146
L3b		3	5	0.774	0.624	0.146–2.654	0.146
M*		8	5	0.346	1.179	0.550–5.372	0.066
**M7**		8	9	0.886	0.931	0.350–2.477	0.055
**M9**		12	8	0.299	1.625	0.646–4.089	0.313
**N9**		10	11	0.915	0.953	0.393–2.310	0.052
N9a		6	9	0.487	0.689	0.239–1.983	0.165
Y		4	2	0.635	2.132	0.385–11.806	0.238
**JT**		3	1	0.580	3.196	0.329–31.057	0.317
T		2	1	0.965	2.117	0.190–23.582	0.142
**RO**		2	2	1.000	1.052	0.146–7.561	0.051
**U**		1	0	0.487	0.994	0.981–1.006	─

* Name of the haplogroup.

**Table 8:** Relationship between haplogroups and schizophrenia among males (significance level = 0.05/24).

Haplogroups	Male Patients (157)	Male Controls (162)	*P*	*OR*	*95% CI*	*Power*
**A**	15	15	0.928	1.035	0.488–2.196	0.052
**B**	19	24	0.478	0.792	0.415–1.511	0.170
**M8**	16	17	0.929	0.968	0. 471–1.990	0.052
M8a	2	7	0.192	0.286	0.058–1.397	0.609
C	6	5	0.719	1.248	0.373–4.174	0.080
Z	7	5	0.520	1.465	0.455–4.718	1.470
CZ	1	0	0.492	0.994	0.981–1.006	─
**D**	31	35	0.682	0.893	0.519–1.536	0.089
**R9**	24	16	0.145	1.647	0.839–3.233	0.543
F	24	16	0.145	1.647	0.839–3.233	0.543
**G**	7	9	0.654	0.793	0.288–2.185	0.097
L3*	0	3	0.257	1.019	0.998–1.041	0.050
L3b	0	3	0.257	1.019	0.998–1.041	0.050
M*	8	8	0.949	1.034	0.378–2.825	0.051
**M7**	14	5	**0.028**	3.074	1.080–8.749	0.867
**M9**	15	11	0.367	1.450	0.644–3.263	0.248
**N9**	5	14	**0.040**	0.348	0.122–0.990	0.830
N9a	4	10	0.114	0.397	0.122–1.295	0.598
Y	1	4	0.386	0.253	0.028–2.291	0.459
**R0**	1	1	1.000	1.032	0.064–16.645	0.050
**R11**	1	2	1.000	0.513	0.046–5.713	0.118
**N1**	1	0	0.492	0.994	0.981–1.006	─
N*	0	1	1.000	1.006	0.994–1.018	0.050
**N2**	0	1	1.000	1.006	0.994–1.018	0.050

* Name of the haplogroup.

## Discussion

Herein, no associations were detected between 4 SNPs and schizophrenia in the northern Chinese Han population. Investigators have shown previously that the C5178A polymorphism was associated with bipolar disorder in the Japanese population [29], and that the 5178C-10389A haplotype was a risk factor for bipolar disorder [15]. It was reported that patients with schizophrenia or bipolar disorder had the same risk-associated genetic variations [30]. Our findings of a lack of an association might be attributable to the different genetic background of population and the small sample size. In the current study, no association was found between the A10398G polymorphism and schizophrenia, which was in agreement with the findings of Zhang et al. [31]. In a study involving a small sample size, it was determined that the G13708A polymorphism was related to schizophrenia; but this relationship was not detected in large sample replicates [32]. In addition, the substitutions of the 3 SNPs (A10398G, G13708A, and C13928G) were predicted to be benign using PolyPhen-2 [33], and these predictions were consistent with our findings. In the northern Han population, the frequencies of 11 haplotypes comprising the 4 SNPs did not significantly differ between the control and patient groups.

It was reported that the total frequency of the 3 haplogroups (M, B, and D) was 0.630 in 11,240 Asian mtDNA sequences [34]; we observed a total frequency of 0.606 in the northern Han Chinese population. In this study, there was no evidence of an association between haplogroups and schizophrenia in the northern Chinese Han population. In Europe, similar results were found [35, 36]. Ueno et al. [37] did not observe an association between haplogroups and schizophrenia among the Japanese population. In a study of the Israeli population, Amar et al. [38] found that the frequency of haplogroup HV (including H and HV *) in a group of patients with schizophrenia was significantly higher than in the control group. However, these authors did not conduct multiple testings or include a suitable control group. Rollins et al. [39] reported that haplogroup HV might be a risk factor for schizophrenia and bipolar disorder, but these authors evaluated a small sample.

Discordant results have been obtained regarding an association of haplogroups with schizophrenia in the Han population. It was found that haplogroup N9a was probably a protective factor for schizophrenia in the Han population [40]. In the Hunan Han population, haplogroup B5a was reported to be a risk factor for schizophrenia [31]. In the study, no proof was found for an association of haplogroups with schizophrenia. In the Han population, discrepancies in observed associations among haplogroups and schizophrenia may be attributable to geographical differences.

Associations between mtDNA polymorphisms and schizophrenia were inconsistent among studies. Several factors may explain these discrepancies. First, the sample sizes of many of these studies were relatively small, so results may be not representative of true relationships between mtDNA and schizophrenia. Second, immigration, environment, intermarriage, and genetic factors affected polymorphisms of mtDNA. Specifically, mtDNA polymorphisms were known to differ among ethnic groups [41–43] and geographic regions [44]. Third, many of these studies lacked multiple testing [35].

In summary, we found on evidence of between-group differences in 4 SNPs (C5178A, A10398G, G13708A, and C13928G) thoughts to be associated with schizophrenia. Moreover, there were no significant differences between control and patient groups in the 11 haplotypes comprising of the 4 SNPs. No evidence was observed for an association of any haplogroup with schizophrenia. The control and coding regions of mtDNA should be both analyzed to detect the relationship between mitochondrial DNA and schizophrenia. Except for the polymorphisms of 4 SNPs (C5178A, A10398G, G13708A, and C13928G) and the control region, the Characteristics of other mtDNA regions and rare variants were not examined. Associations between other variations in coding region and schizophrenia were not detected in this study. The relationship between mtDNA and schizophrenia requires further study.

## Supporting information

S1 Table MtDNA haplogroups and haplotypes among control-group participants (n = 326). (XLSX)

S2 Table MtDNA haplogroups and haplotypes among patients with schizophrenia (n = 313). (XLSX)

## References

[1] Sullivan PF. The genetics of schizophrenia. PLoS Med. 2005;2(7):e212 doi: 10.1371/journal.pmed.0020212 ; PubMed Central PMCID: PMC1181880. 16033310 10.1371/journal.pmed.0020212 PMC1181880

[2] Goldstein JM, Faraone SV, Chen WJ, Tsuang MT. Gender and the familial risk for schizophrenia. Disentangling confounding factors. Schizophr Res. 1992;7(2):135–40. . 1515374 10.1016/0920-9964(92)90043-5

[3] Dimauro S, Davidzon G. Mitochondrial DNA and disease. Ann Med. 2005;37(3):222–32. doi: 10.1080/07853890510007368 . 16019721 10.1080/07853890510007368

[4] Taanman JW. The mitochondrial genome: structure, transcription, translation and replication. Biochim Biophys Acta. 1999;1410(2):103–23. . 10076021 10.1016/s0005-2728(98)00161-3

[5] Ben-Shachar D, Laifenfeld D. Mitochondria, synaptic plasticity, and schizophrenia. Int Rev Neurobiol. 2004;59:273–96. doi: 10.1016/S0074-7742(04)59011-6 . 15006492 10.1016/S0074-7742(04)59011-6

[6] Pietka G, Kukwa W, Bartnik E, Scinska A, Czarnecka AM. [Mitochondrial DNA mutations in the pathogenesis in the head and neck squamous cell carcinoma]. Otolaryngol Pol. 2008;62(2):158–64. doi: 10.1016/S0030-6657(08)70233-6 . 18637439 10.1016/S0030-6657(08)70233-6

[7] Ishikawa K, Koshikawa N, Takenaga K, Nakada K, Hayashi J. Reversible regulation of metastasis by ROS-generating mtDNA mutations. Mitochondrion. 2008;8(4):339–44. doi: 10.1016/j.mito.2008.07.006 . 18727959 10.1016/j.mito.2008.07.006

[8] Diop AG, de Boer HM, Mandlhate C, Prilipko L, Meinardi H. The global campaign against epilepsy in Africa. Acta Trop. 2003;87(1):149–59. . 12781390 10.1016/s0001-706x(03)00038-x

[9] Ben-Shachar D, Zuk R, Gazawi H, Reshef A, Sheinkman A, Klein E. Increased mitochondrial complex I activity in platelets of schizophrenic patients. Int J Neuropsychopharmacol. 1999;2(4):245–53. doi: 10.1017/S1461145799001649 . 11285140 10.1017/S1461145799001649

[10] Amberger JS, Bocchini CA, Schiettecatte F, Scott AF, Hamosh A. OMIM.org: Online Mendelian Inheritance in Man (OMIM(R)), an online catalog of human genes and genetic disorders. Nucleic acids research. 2015;43(Database issue):D789–98. doi: 10.1093/nar/gku1205 ; PubMed Central PMCID: PMC4383985. 25428349 10.1093/nar/gku1205 PMC4383985

[11] Levine RL, Mosoni L, Berlett BS, Stadtman ER. Methionine residues as endogenous antioxidants in proteins. Proc Natl Acad Sci U S A. 1996;93(26):15036–40. ; PubMed Central PMCID: PMC26351. 8986759 10.1073/pnas.93.26.15036 PMC26351

[12] Takagi K, Yamada Y, Gong JS, Sone T, Yokota M, Tanaka M. Association of a 5178C—>A (Leu237Met) polymorphism in the mitochondrial DNA with a low prevalence of myocardial infarction in Japanese individuals. Atherosclerosis. 2004;175(2):281–6. doi: 10.1016/j.atherosclerosis.2004.03.008 . 15262184 10.1016/j.atherosclerosis.2004.03.008

[13] Nijiati M, Saidaming A, Qiao J, Cheng Z, Qiu C, Sun Y. GNB3, eNOS, and mitochondrial DNA polymorphisms correlate to natural longevity in a Xinjiang Uygur population. PLoS One. 2013;8(12):e81806 doi: 10.1371/journal.pone.0081806 ; PubMed Central PMCID: PMC3869651. 24376503 10.1371/journal.pone.0081806 PMC3869651

[14] Brown MD, Shoffner JM, Kim YL, Jun AS, Graham BH, Cabell MF, et al Mitochondrial DNA sequence analysis of four Alzheimer's and Parkinson's disease patients. Am J Med Genet. 1996;61(3):283–9. doi: 10.1002/(SICI)1096-8628(19960122)61:3<283::AID-AJMG15>3.0.CO;2-P . 8741876 10.1002/(SICI)1096-8628(19960122)61:3<283::AID-AJMG15>3.0.CO;2-P

[15] Kato T, Kunugi H, Nanko S, Kato N. Mitochondrial DNA polymorphisms in bipolar disorder. J Affect Disord. 2001;62(3):151–64. . 11223103 10.1016/s0165-0327(99)00173-1

[16] Liou CW, Chen JB, Tiao MM, Weng SW, Huang TL, Chuang JH, et al Mitochondrial DNA coding and control region variants as genetic risk factors for type 2 diabetes. Diabetes. 2012;61(10):2642–51. doi: 10.2337/db11-1369 ; PubMed Central PMCID: PMC3447893. 22891220 10.2337/db11-1369 PMC3447893

[17] Pezzotti A, Kraft P, Hankinson SE, Hunter DJ, Buring J, Cox DG. The mitochondrial A10398G polymorphism, interaction with alcohol consumption, and breast cancer risk. PLoS One. 2009;4(4):e5356 doi: 10.1371/journal.pone.0005356 ; PubMed Central PMCID: PMC2668794. 19390621 10.1371/journal.pone.0005356 PMC2668794

[18] Jiang H, Zhao H, Xu H, Hu L, Wang W, Wei Y, et al Peripheral blood mitochondrial DNA content, A10398G polymorphism, and risk of breast cancer in a Han Chinese population. Cancer Sci. 2014;105(6):639–45. doi: 10.1111/cas.12412 ; PubMed Central PMCID: PMC4317893. 24703408 10.1111/cas.12412 PMC4317893

[19] Fachal L, Mosquera-Miguel A, Pastor P, Ortega-Cubero S, Lorenzo E, Oterino-Duran A, et al No evidence of association between common European mitochondrial DNA variants in Alzheimer, Parkinson, and migraine in the Spanish population. Am J Med Genet B Neuropsychiatr Genet. 2015;168B(1):54–65. doi: 10.1002/ajmg.b.32276 . 25349034 10.1002/ajmg.b.32276

[20] Zsurka G, Kalman J, Csaszar A, Rasko I, Janka Z, Venetianer P. No mitochondrial haplotype was found to increase risk for Alzheimer's disease. Biol Psychiatry. 1998;44(5):371–3. . 9755361 10.1016/s0006-3223(97)00461-7

[21] Lobo A, Perez-Echeverria MJ, Artal J. Validity of the scaled version of the General Health Questionnaire (GHQ-28) in a Spanish population. Psychological medicine. 1986;16(1):135–40. . 3961039 10.1017/s0033291700002579

[22] Roig B, Virgos C, Franco N, Martorell L, Valero J, Costas J, et al The discoidin domain receptor 1 as a novel susceptibility gene for schizophrenia. Mol Psychiatry. 2007;12(9):833–41. doi: 10.1038/sj.mp.4001995 . 17440435 10.1038/sj.mp.4001995

[23] Pereira J, Rui N, Forat S, Huckenbeck W, Olek K. MtDNA typing of single-sperm cells isolated by micromanipulation. Forensic Science International Genetics. 2012;6(2):228–35. doi: 10.1016/j.fsigen.2011.05.005 21680273 10.1016/j.fsigen.2011.05.005

[24] Andrews RM, Kubacka I, Chinnery PF, Lightowlers RN, Turnbull DM, Howell N. Reanalysis and revision of the Cambridge reference sequence for human mitochondrial DNA. Nat Genet. 1999;23(2):147 doi: 10.1038/13779 . 10508508 10.1038/13779

[25] Fan L, Yao YG. An update to MitoTool: using a new scoring system for faster mtDNA haplogroup determination. Mitochondrion. 2013;13(4):360–3. doi: 10.1016/j.mito.2013.04.011 . 23632257 10.1016/j.mito.2013.04.011

[26] Chu Q, Luo X, Zhan X, Ren Y, Pang H. Female genetic distribution bias in mitochondrial genome observed in Parkinson's Disease patients in northern China. Scientific reports. 2015;5:17170 doi: 10.1038/srep17170 ; PubMed Central PMCID: PMC4658531. 26602989 10.1038/srep17170 PMC4658531

[27] Dupont WD, Plummer WD Jr. Power and sample size calculations for studies involving linear regression. Controlled clinical trials. 1998;19(6):589–601. . 9875838 10.1016/s0197-2456(98)00037-3

[28] Ingman M, Gyllensten U. mtDB: Human Mitochondrial Genome Database, a resource for population genetics and medical sciences. Nucleic acids research. 2006;34(Database issue):D749–51. doi: 10.1093/nar/gkj010 ; PubMed Central PMCID: PMC1347373. 16381973 10.1093/nar/gkj010 PMC1347373

[29] Kato T, Kunugi H, Nanko S, Kato N. Association of bipolar disorder with the 5178 polymorphism in mitochondrial DNA. Am J Med Genet. 2000;96(2):182–6. . 10893494

[30] Guan F, Lin H, Chen G, Li L, Chen T, Liu X, et al Evaluation of association of common variants in HTR1A and HTR5A with schizophrenia and executive function. Sci Rep. 2016;6:38048 doi: 10.1038/srep38048 ; PubMed Central PMCID: PMC5126681. 27897266 10.1038/srep38048 PMC5126681

[31] Zhang W, Tang J, Zhang AM, Peng MS, Xie HB, Tan L, et al A matrilineal genetic legacy from the last glacial maximum confers susceptibility to schizophrenia in Han Chinese. Journal of genetics and genomics = Yi chuan xue bao. 2014;41(7):397–407. doi: 10.1016/j.jgg.2014.05.004 . 25064678 10.1016/j.jgg.2014.05.004

[32] Bamne MN, Talkowski ME, Moraes CT, Manuck SB, Ferrell RE, Chowdari KV, et al Systematic association studies of mitochondrial DNA variations in schizophrenia: focus on the ND5 gene. Schizophr Bull. 2008;34(3):458–65. doi: 10.1093/schbul/sbm100 ; PubMed Central PMCID: PMC2632438. 17898419 10.1093/schbul/sbm100 PMC2632438

[33] Adzhubei I, Jordan DM, Sunyaev SR. Predicting functional effect of human missense mutations using PolyPhen-2. Current protocols in human genetics. 2013;Chapter 7:Unit7 20. doi: 10.1002/0471142905.hg0720s76 ; PubMed Central PMCID: PMC4480630. 23315928 10.1002/0471142905.hg0720s76 PMC4480630

[34] Lott MT, Leipzig JN, Derbeneva O, Xie HM, Chalkia D, Sarmady M, et al mtDNA Variation and Analysis Using Mitomap and Mitomaster. Current protocols in bioinformatics. 2013;44:1 23 1–6. doi: 10.1002/0471250953.bi0123s44 ; PubMed Central PMCID: PMC4257604. 25489354 10.1002/0471250953.bi0123s44 PMC4257604

[35] Mosquera-Miguel A, Torrell H, Abasolo N, Arrojo M, Paz E, Ramos-Rios R, et al No evidence that major mtDNA European haplogroups confer risk to schizophrenia. Am J Med Genet B Neuropsychiatr Genet. 2012;159B(4):414–21. doi: 10.1002/ajmg.b.32044 . 22467472 10.1002/ajmg.b.32044

[36] Torrell H, Salas A, Abasolo N, Moren C, Garrabou G, Valero J, et al Mitochondrial DNA (mtDNA) variants in the European haplogroups HV, JT, and U do not have a major role in schizophrenia. American journal of medical genetics Part B, Neuropsychiatric genetics: the official publication of the International Society of Psychiatric Genetics. 2014;165B(7):607–17. doi: 10.1002/ajmg.b.32264 . 25132006 10.1002/ajmg.b.32264

[37] Ueno H, Nishigaki Y, Kong QP, Fuku N, Kojima S, Iwata N, et al Analysis of mitochondrial DNA variants in Japanese patients with schizophrenia. Mitochondrion. 2009;9(6):385–93. doi: 10.1016/j.mito.2009.06.003 . 19563917 10.1016/j.mito.2009.06.003

[38] Amar S, Shamir A, Ovadia O, Blanaru M, Reshef A, Kremer I, et al Mitochondrial DNA HV lineage increases the susceptibility to schizophrenia among Israeli Arabs. Schizophrenia Research. 2007;94(1–3):354–8. doi: 10.1016/j.schres.2007.04.020 17566709 10.1016/j.schres.2007.04.020

[39] Rollins B, Martin MV, Sequeira PA, Moon EA, Morgan LZ, Watson SJ, et al Mitochondrial variants in schizophrenia, bipolar disorder, and major depressive disorder. PLoS One. 2009;4(3):e4913 doi: 10.1371/journal.pone.0004913 ; PubMed Central PMCID: PMC2654519. 19290059 10.1371/journal.pone.0004913 PMC2654519

[40] Wang GX, Zhang Y, Zhang YT, Dong YS, Lv ZW, Sun M, et al Mitochondrial haplogroups and hypervariable region polymorphisms in schizophrenia: a case-control study. Psychiatry Res. 2013;209(3):279–83. doi: 10.1016/j.psychres.2013.01.001 . 23374981 10.1016/j.psychres.2013.01.001

[41] Zhou HY, Wang HW, Tan SN, Chen Y, Wang WL, Tao HX, et al Genetic affinities of central China populations. Genetics and molecular research: GMR. 2014;13(1):616–25. doi: 10.4238/2014.January.28.7 . 24615027 10.4238/2014.January.28.7

[42] Chen F, Yin CY, Qian XQ, Fan HT, Deng YJ, Zhang YD, et al Single nucleotide polymorphisms of mitochondrial DNA HVS-I and HVS-II in Chinese Bai ethnic group. Electrophoresis. 2015;36(6):930–6. doi: 10.1002/elps.201400493 . 25488882 10.1002/elps.201400493

[43] Xu FL, Yao J, Ding M, Shi ZS, Wu X, Zhang JJ, et al Characterization of mitochondrial DNA polymorphisms in the Han population in Liaoning Province, Northeast China. Mitochondrial DNA Part A, DNA mapping, sequencing, and analysis. 2017:1–9. doi: 10.1080/24701394.2016.1275597 . 28093929 10.1080/24701394.2016.1275597

[44] Yao YG, Kong QP, Bandelt HJ, Kivisild T, Zhang YP. Phylogeographic differentiation of mitochondrial DNA in Han Chinese. Am J Hum Genet. 2002;70(3):635–51. doi: 10.1086/338999 ; PubMed Central PMCID: PMC384943. 11836649 10.1086/338999 PMC384943

